# Analysis of Diagnoses, Symptoms, Medications, and Admissions Among Patients With Cancer Presenting to Emergency Departments

**DOI:** 10.1001/jamanetworkopen.2019.0979

**Published:** 2019-03-22

**Authors:** Jeffrey M. Caterino, David Adler, Danielle D. Durham, Sai-Ching Jim Yeung, Matthew F. Hudson, Aveh Bastani, Steven L. Bernstein, Christopher W. Baugh, Christopher J. Coyne, Corita R. Grudzen, Daniel J. Henning, Adam Klotz, Troy E. Madsen, Daniel J. Pallin, Cielito C. Reyes-Gibby, Juan Felipe Rico, Richard J. Ryan, Nathan I. Shapiro, Robert Swor, Arvind Venkat, Jason Wilson, Charles R. Thomas, Jason J. Bischof, Gary H. Lyman

**Affiliations:** 1 Department of Internal Medicine, The Ohio State University Wexner Medical Center, Columbus; 2 Department of Emergency Medicine, The Ohio State University Wexner Medical Center, Columbus; 3 Department of Emergency Medicine, University of Rochester, Rochester, New York; 4 Healthcare Delivery Research Program, Division of Cancer Control and Population Sciences, National Cancer Institute, Rockville, Maryland; 5 Department of Endocrine Neoplasia and Hormonal Disorders, The University of Texas MD Anderson Cancer Center, Houston; 6 Department of Emergency Medicine, The University of Texas MD Anderson Cancer Center, Houston; 7 Greenville Health System Cancer Institute, Greenville, South Carolina; 8 Department of Emergency Medicine, William Beaumont Hospital–Troy Campus, Troy, Michigan; 9 Department of Emergency Medicine, Yale School of Medicine, New Haven, Connecticut; 10 Department of Emergency Medicine, Brigham and Women’s Hospital, Boston, Massachusetts; 11 Department of Emergency Medicine, University of California, San Diego; 12 Ronald O. Perelman Department of Emergency Medicine and Population Health, New York University School of Medicine, New York; 13 Department of Emergency Medicine, University of Washington, Seattle; 14 Department of Medicine, Memorial Sloan Kettering Cancer Center, New York, New York; 15 Division of Emergency Medicine, University of Utah, Salt Lake City; 16 Department of Pediatrics, University of South Florida Morsani College of Medicine, Tampa; 17 Department of Emergency Medicine, University of Cincinnati, Cincinnati, Ohio; 18 Department of Emergency Medicine, Beth Israel Deaconness Medical Center, Boston, Massachusetts; 19 Department of Emergency Medicine, William Beaumont Hospital, Royal Oak, Michigan; 20 Department of Emergency Medicine, Allegheny Health Network, Pittsburgh, Pennsylvania; 21 Department of Emergency Medicine, University of South Florida Morsani College of Medicine, Tampa; 22 Department of Radiation Medicine, Knight Cancer Institute, Oregon Health & Sciences University, Portland; 23 Hutchinson Institute for Cancer Outcomes Research, Fred Hutchinson Cancer Research Center, Department of Medicine, University of Washington School of Medicine, Seattle

## Abstract

**Importance:**

Better understanding of the emergency care needs of patients with cancer will inform outpatient and emergency department (ED) management.

**Objective:**

To provide a benchmark description of patients who present to the ED with active cancer.

**Design, Setting, and Participants:**

This multicenter prospective cohort study included 18 EDs affiliated with the Comprehensive Oncologic Emergencies Research Network (CONCERN). Of 1564 eligible patients, 1075 adults with active cancer were included from February 1, 2016, through January 30, 2017. Data were analyzed from February 1 through August 1, 2018.

**Main Outcomes and Measures:**

The proportion of patients reporting symptoms (eg, pain, nausea) before and during the ED visit, ED and outpatient medications, most common diagnoses, and suspected infection as indicated by ED antibiotic administration. The proportions observed, admitted, and with a hospital length of stay (LOS) of no more than 2 days were identified.

**Results:**

Of 1075 participants, mean (SD) age was 62 (14) years, and 51.8% were female. Seven hundred ninety-four participants (73.9%; 95% CI, 71.1%-76.5%) had undergone cancer treatment in the preceding 30 days; 674 (62.7%; 95% CI, 59.7%-65.6%) had advanced or metastatic cancer; and 505 (47.0%; 95% CI, 43.9%-50.0%) were 65 years or older. The 5 most common ED diagnoses were symptom related. Of all participants, 82 (7.6%; 95% CI, 6.1%-9.4%) were placed in observation and 615 (57.2%; 95% CI, 54.2%-60.2%) were admitted; 154 of 615 admissions (25.0%; 95% CI, 21.7%-28.7%) had an LOS of 2 days or less (median, 3 days; interquartile range, 2-6 days). Pain during the ED visit was present in 668 patients (62.1%; 95% CI, 59.2%-65.0%; mean [SD] pain score, 6.4 [2.6] of 10.0) and in 776 (72.2%) during the prior week. Opioids were administered in the ED to 228 of 386 patients (59.1%; 95% CI, 18.8%-23.8%) with moderate to severe ED pain. Outpatient opioids were prescribed to 368 patients (47.4%; 95% CI, 3.14%-37.2%) of those with pre-ED pain, including 244 of 428 (57.0%; 95% CI, 52.2%-61.8%) who reported quite a bit or very much pain. Nausea in the ED was present in 336 (31.3%; 95% CI, 28.5%-34.1%); of these, 160 (47.6%; 95% CI, 12.8%-17.1%) received antiemetics in the ED. Antibiotics were administered in the ED to 285 patients (26.5%; 95% CI, 23.9%-29.2%). Of these, 209 patients (73.3%; 95% CI, 17.1%-21.9%) were admitted compared with 427 of 790 (54.1%; 95% CI, 50.5%-57.6%) not receiving antibiotics.

**Conclusions and Relevance:**

This initial prospective, multicenter study profiling patients with cancer who were treated in the ED identifies common characteristics in this patient population and suggests opportunities to optimize care before, during, and after the ED visit. Improvement requires collaboration between specialists and emergency physicians optimizing ED use, improving symptom control, avoiding unnecessary hospitalizations, and appropriately stratifying risk to ensure safe ED treatment and disposition of patients with cancer.

## Key Points

**Question:**

What are the characteristics of patients with active cancer presenting to US emergency departments?

**Findings:**

In this multicenter cohort study of 1075 adult patients with active cancer in the Comprehensive Oncologic Emergencies Research Network (CONCERN), patients commonly presented with symptoms such as pain (62.1%) and nausea (31.3%), were frequently treated for potential infection (26.5%), and were admitted (57.2%; 25.0% for <2 days) or placed in observation (7.6%).

**Meaning:**

Opportunities for improving emergency department care for patients with cancer include establishing protocols and processes for prompt and appropriate symptom control, creating improved risk stratification tools, and improving outpatient management to prevent ED visits.

## Teaser

This multicenter cohort study identifies the clinical characteristics and needs of adults with active cancer who present to emergency departments (EDs).

## Introduction

Patients with cancer account for more than 4.5 million US emergency department (ED) visits annually.^1,2^ This estimate, derived from ED diagnostic codes, likely underestimates the true annual incidence of cancer-related ED visits among the 15.5 million US residents with cancer.^3^ Approximately two-thirds of ED visits by patients with cancer result in hospital admission,^4,5^ affirming the ED as a critical portal addressing acute illness issues and the continuum of cancer management.

Oncologic emergency medicine has traditionally focused on the diagnosis and management of the adverse effects of cancer treatment (eg, febrile neutropenia, thrombosis, tumor lysis syndrome, or hypercalcemia) and patient-centered care issues (eg, symptom control and quality of life).^6^ Advances in oncology treatments present challenges of new adverse effects and an expanding spectrum of cancer-treatment-related emergencies.^7^ Symptom-driven issues are the most common complaints in patients with cancer visiting EDs, including pain and respiratory and gastrointestinal tract issues.^2,4^ However, a knowledge gap exists for providing optimal cancer care in the ED.^8^

Retrospective studies have described characteristics of patients with cancer who present to the ED.^2,4,9,10,11,12^ Currently available data, gleaned from retrospective registries or surveys, lack granularity regarding ED course, functional status, symptom inventories, and other variables critical to understanding ED visits by patients with cancer.^4^ To address this knowledge deficiency, the National Cancer Institute sponsors the Comprehensive Oncologic Emergencies Research Network (CONCERN), a multicenter research consortium including representatives from oncology and emergency medicine.^13,14^ The National Cancer Institute has expressed interest in clarifying cancer-related use of emergency care, risk stratification, and outcome improvement.^15^ CONCERN aspires to accelerate knowledge generation as well as design, execution, and translation of oncologic emergency medicine research. CONCERN’s focus areas include but are not limited to epidemiology, febrile neutropenia and other infections, acute symptom control, venous thromboembolism, and palliative care.^8,14^

This study addresses these essential high-priority goals and interests. The primary objective of this study is to establish a benchmark description of the population of patients with active cancer presenting to CONCERN EDs.

## Methods

### Study Design, Setting, and Participants

We conducted a multicenter prospective observational cohort study in 18 CONCERN-affiliated EDs from March 1, 2016, through January 30, 2017, with institutional review board approval at each site. All participants provided written informed consent, and no incentive was provided. This study adhered to the Strengthening the Reporting of Observational Studies in Epidemiology (STROBE) reporting guidelines.^16^ The study sites were academic EDs with a mean annual volume of 71 886 patients and a mean admission rate of 30%. The median proportion of ED visits of patients with active cancer was 7.5% (interquartile range [IQR], 4.0%-15.0%). Seventeen sites were urban, and 13 were affiliated with National Cancer Institute–designated comprehensive cancer centers (a complete list appears in eTable 1 in the Supplement).

Study participants consisted of a nonconsecutive sample of adults (aged ≥18 years) with active cancer who presented in the ED when research staff were available. Availability varied by site, but generally consisted of weekdays 7 am until 8 to 11 pm with some weekend and overnight (11 pm-7 am) availability. Active cancer was defined by (1) ongoing (or within 12 months) antineoplastic therapy (radiotherapy, chemotherapy, cancer-related surgery, or other), (2) previously identified or ED physician–identified cancer recurrence or metastasis, or (3) cancer-related symptoms.^17^ Exclusion criteria included pregnancy, incarceration, psychiatric chief complaint, primary evaluation as a trauma response, non–English speaking, previous enrollment, or too ill or otherwise unable to participate in survey administration.

### Study Procedures and Measures

Research staff administered a patient questionnaire in the ED. Although the patient was the primary responder, family and caregivers could assist with survey completion. After a minimum of 1 hour of training, study staff collected data from medical record review 30 days after enrollment using standardized electronic forms and a data dictionary.^18^ Records reviewed included the ED and inpatient records and subsequent outpatient notes during the 30 days.

Survey variables included demographics, cancer type, cancer therapy within the preceding 30 days (chemotherapy, targeted therapy, systemic corticosteroids, radiotherapy, or cancer-related surgery), and outpatient medications by pharmacologic class (eg, pain medication classes included nonsteroidal anti-inflammatory drugs, acetaminophen, tramadol hydrochloride, short- or long-acting opioids, and/or other). We defined advanced cancer based on the protocol of Kandarian et al^19^ as metastatic, recurrent, unresectable, locally advanced, stage III, or stage IV disease. Advanced directives were reported as not present, do not resuscitate, do not intubate, comfort care only, or other. We recorded receipt of palliative care or hospice services within the last 30 days. We collected data for Eastern Cooperative Oncology Group performance status^20^ and the Functional Assessment of Cancer Therapy–General.^21^ Participants reported their highest measured temperature in the prior 24 hours and symptoms in the ED, including any pain, chest pain, shortness of breath, nausea, abdominal pain, or urinary tract symptoms.

Medical record review data included emergency severity index^22^; ED vital signs; comorbidities sufficient to calculate the Charlson comorbidity index^23^; primary cancer type; cancer grade and stage; recent cancer-related therapies; outpatient- and ED-administered pain medications, nausea medications, and antibiotics; ED diagnoses and disposition; inpatient diagnoses; additional ED visits and/or hospitalizations within 30 days; 30-day mortality; ED laboratory and imaging results; hospital length of stay (LOS); and presence of advance directives. We recorded patients’ initial ED pain scores on a 10-point scale categorized as none (0), mild (1-4), moderate (5-6), or severe (7-10).^24^ We recorded as many as 4 ED diagnoses using the *International Statistical Classification of Diseases and Related Health Problems, Tenth Revision*.

### Study Size

We anticipated enrolling as many as 70 participants per site (ie, a maximum of 1260 participants). To allow for potential underrecruitment, we calculated that a minimum of 840 patients (two-thirds of enrollment target) would provide sufficiently precise estimates such that proportions would have exact 95% CIs with a 2-sided width of less than 5%, which would allow precise estimates to clarify salient characteristics of the population presenting to an ED with active cancer.

### Statistical Analysis

Data were analyzed from February 1 through August 1, 2018, using SAS software (version 9.4; SAS Institute, Inc) and included descriptive statistics. We calculated means with SDs, medians with IQR, and proportions with 95% CIs. Normality was tested using the Shapiro Wilk test with a 2-sided *P* < .05 considered significant. We measured interrater reliability of the medical record review using the Cohen κ statistic through review of a random sample of 10% of medical records at each site by a second abstractor.

## Results

For enrollment, 1562 of 2337 screened patients were eligible, and 1075 of these (68.8%; 95% CI, 66%-71%) consented to participate (eFigure in the Supplement). Nonenrollees did not differ from enrollees by sex, age, or day of week screened. Study population characteristics are summarized in Table 1. Mean (SD) age was 62 (14) years, with 505 (47.0%; 95% CI, 43.9%-50.0%) 65 years or older and 99 (9.2%; 95% CI, 7.5%-11.1%) 80 years or older (557 female [51.8%; 95% CI, 48.8%-54.8%] and 518 male [48.2%; 95% CI, 45.2%-51.2%]). Four hundred thirty patients (40.0%; 95% CI, 37.0%-43.0%) had an emergency severity index of 2; 542 (50.4%; 95% CI, 47.4%-53.4%) had an emergency severity index of 3. Mean (SD) Charlson comorbidity score was 4.2 (3.1). The 5 top ED diagnoses were symptom related, including abdominal pain (100 [9.3%; 95% CI, 7.6%-11.2%]), fever (87 [8.1%; 95% CI, 6.5%-9.9%]), breathing abnormalities (77 [7.2%; 95% CI, 5.7%-8.9%]), nausea and vomiting (60 [5.6%; 95% CI, 4.3%-7.1%]), and throat and chest pain (51 [4.7%; 95% CI, 3.6%-6.2%]) (Table 2). Other symptom- and pain-related diagnoses were common.

**Table 1.** Characteristics of Patients With Active Cancer Presenting to the ED^a^

Variable	No. of Patients (N = 1075)	Proportion, % (95% CI)
Age ≥65 y	505	47.0 (43.9-50.0)
Female	557	51.8 (48.8-54.8)
Race		
Black or African American	129	12.0 (10.1-14.1)
White	847	78.8 (76.2-81.2)
Other	38	3.5 (2.5-4.8)
Missing	61	5.7 (4.4-7.2)
Ethnicity		
Hispanic/Latino	76	7.1 (5.6-8.8)
Not Hispanic/Latino	977	90.9 (94.0-96.6)
Missing	22	2.0 (1.3-3.1)
Marital status		
Married or domestic partnership	639	59.4 (56.4-62.4)
Never married	151	14.0 (12.0-16.3)
Divorced or separated	153	14.2 (12.2-16.5)
Widowed	123	11.4 (9.6-13.5)
Missing	9	0.8 (0.4-1.6)
Educational attainment		
Not high school graduate	85	7.9 (6.4-9.7)
High school graduate or equivalent	270	25.1 (22.5-27.8)
Some college or associate’s degree	283	26.3 (23.7-29.1)
Bachelor’s degree	225	20.9 (18.5-23.5)
Graduate or professional degree	195	18.1 (15.9-20.6)
Missing	17	1.6 (0.9-2.5)
ED arrival on a weekend		
Yes	135	12.6 (10.6-14.7)
No	929	86.4 (84.2-88.4)
Missing	11	1.0 (0.5-1.8)
ED arrival shift		
Daytime (7 am-3 pm)	641	59.6 (56.6-62.6)
Evening (3 pm-11 pm)	329	30.6 (27.8-33.4)
Night (11 pm-7 am)	94	8.7 (7.1-10.6)
Missing	11	1.0 (0.5-1.8)
Mode of ED arrival		
EMS	244	22.7 (20.2-25.3)
Non-EMS	702	65.3 (62.4-68.1)
Missing	129	12.0 (10.1-14.1)
Emergency severity index^b^		
1 (Severely unstable)	10	0.9 (0.4-1.7)
2 (Potentially unstable)	430	40.0 (37.0-43.0)
3 (Stable-urgent)	542	50.4 (47.4-53.4)
4 (Stable-less urgent)	20	1.9 (1.1-2.8)
5 (Nonurgent)	6	0.6 (0.2-1.2)
6 (Not documented)	57	5.3 (4.0-6.8)
Missing	10	0.9 (0.4-1.7)
ECOG score		
0 (Asymptomatic)	302	28.1 (25.4-30.9)
1 (Symptomatic, but completely ambulatory)	324	30.1 (27.4-33.0)
2 (Symptomatic, <50% of time in bed during the day)	202	18.8 (16.5-21.2)
3 (Symptomatic, >50% of time in bed, but not bed bound)	199	18.5 (16.2-21.0)
4 (Bed bound)	32	3.0 (2.0-4.2)
5 (Death)	0	0.0 (0-0.3)
Missing	16	1.5 (0.8-2.4)
ED disposition		
Admission to regular floor	504	46.9 (4.4-5.0)
Admission to step-down unit	66	6.1 (4.8-7.7)
Admission to ICU	45	4.2 (3.1-5.5)
Discharge home	342	31.8 (2.9-3.5)
Discharge to ECF or rehabilitation facility	4	0.4 (0.1-0.9)
Transfer to another facility	20	1.9 (1.1-2.8)
Died in ED	0	0.0 (0.0-0.3)
ED observation	70	6.5 (5.1-8.2)
Hospital observation	12	1.1 (0.6-1.9)
Missing	12	1.1 (0.6-1.9)
Hospital admission within 30 d after index encounter		
Yes	274	25.5 (22.9-28.2)
No	790	73.5 (70.7-76.1)
Missing	11	1.0 (0.5-1.8)
ED revisit within 30 d		
Yes	286	26.6 (24.0-29.4)
No	778	72.4 (69.6-75.0)
Missing	11	1.0 (0.5-1.8)
30-d mortality		
Yes	62	5.8 (4.4-7.3)
No	965	89.8 (87.8-91.5)
Missing	48	4.5 (3.3-5.9)

Abbreviations: ECF, extended care facility; ECOG, Eastern Collaborative Oncology Group; ED, emergency department; EMS, emergency medical services; ICU, intensive care unit.

^a^ Mean (SD) age of the study group was 62 (14) years; mean (SD) Charlson comorbidity index, 4.2 (3.1) (calculated from ED documentation and patient problem list, not from diagnoses coded for that specific visit); and median hospital length of stay, 3 days (interquartile range, 2-6 days).

^b^ A score of 1 indicates should be seen immediately by a physician; 2, within 10 minutes; and 3, within 30 minutes.

**Table 2.** Most Common ED Diagnoses Among 1075 Patients With Active Cancer

*ICD-10-CM* Code	*ICD-10-CM* Category	Frequency, No. (%) [95% CI]
R10	Abdominal and pelvic pain	100 (9.3) [7.6-11.2]
R50	Fever of other and unknown origin	87 (8.1) [6.5-9.9]
R06	Abnormalities of breathing	77 (7.2) [5.7-8.9]
R11	Nausea and vomiting	60 (5.6) [4.3-7.1]
R07	Pain in throat and chest	51 (4.7) [3.6-6.2]
D64	Other anemias	47 (4.4) [3.2-5.8]
E87	Other disorders of fluid, electrolytes, and acid-base balance	47 (4.4) [3.2-5.8]
R53	Malaise and fatigue	45 (4.2) [3.1-5.6]
E86	Volume depletion	43 (4.0) [2.9-5.4]
I26	Pulmonary embolism	39 (3.6) [2.6-4.9]
J18	Pneumonia, unspecified organism	39 (3.6) [2.6-4.9]
D70	Neutropenia	37 (3.4) [2.4-4.7]
C34	Malignant neoplasm of bronchus and lung	36 (3.3) [2.4-4.6]
N39	Other disorders of urinary system	36 (3.3) [2.4-4.6]
R19	Other symptoms and signs involving the digestive system and abdomen	36 (3.3) [2.4-4.6]
C79	Secondary malignant neoplasm of other and unspecified sites	35 (3.3) [2.3-4.5]
M54	Dorsalgia (eg, radiculopathy, sciatica)	33 (3.1) [2.1-4.3]
G89	Pain, not elsewhere classified	28 (2.6) [1.7-3.7]
M79	Other and unspecified soft tissue disorders, not elsewhere classified (ie, nonspecific pain)	26 (2.4) [1.6-3.5]
R55	Syncope and collapse	26 (2.4) [1.6-3.5]

Abbreviations: ED, emergency department; *ICD-10-CM*, *International Statistical Classification of Diseases, Tenth Revision, Clinical Modification*.

Emergency department disposition included admission for 615 participants (57.2%; 95% CI, 54.2%-60.2%) and observation status for 82 (7.6%; 95% CI, 6.1%-9.4%) (Table 1). Of those admitted, mean (SD) LOS was 5.0 (5.6) days and median LOS was 3 days (IQR, 2-6 days); 154 of 615 admissions (25.0%; 95% CI, 21.7%-28.7%) had an LOS of 2 days or less, including 91 participants (14.8%; 95% CI, 12.1%-17.8%) with an LOS of 0 to 1 day and 63 (10.0%; 95% CI, 8.0%-12.9%) with an LOS of 2 days. Sixty-two participants (5.8%) died within 30 days of first presentation to the ED. Thirty-day mortality included 62 participants (5.8%; 95% CI, 4.4%-7.3%). Within 30 days of the enrollment visit, 286 participants (26.6%; 95% CI, 24.0%-29.45) had an ED revisit and 274 (25.5%; 95% CI, 22.9%-28.2%) had a separate hospital admission, including a second hospital admission in 180 of the 635 admitted during the initial enrollment visit. Forty-four participants (4.1%; 95% CI, 3.0%-49.7%) had more than 1 readmission.

The most common cancer types were gastrointestinal tract (220 [20.5%; 95% CI, 18.1%-23.0%]), lung (139 [12.9%; 95% CI, 11.0%-15.1%]), hematologic (128 [11.9%; 95% CI, 10.0%-14.0%]), and breast (118 [11.0%; 95% CI, 11.0%-15.1%]) (Table 3). Most participants had received treatment for cancer in the last 30 days (794 [73.9%; 71.1%-76.4%]). Although advanced or metastatic cancer was present in 674 (62.7%; 95% CI, 59.7%-65.6%), advance directives were present in 502 (46.7%; 95% CI, 43.7%-49.7%) including 193 (18.0%; 95% CI, 15.7%-20.4%) with a full-code directive. Twenty participants (1.9%; 95% CI, 1.1%-2.8%) were receiving hospice care and 86 (8.0%; 95% CI, 6.4%-9.8%) were receiving palliative care.

**Table 3.** Cancer Type and Treatment in 1075 Patients With Cancer Presenting to the Emergency Department^a^

Characteristic	Frequency, No. (%) [95% CI]
Primary cancer type	
Gastrointestinal tract	220 (20.5) [18.1-23.0]
Esophageal	26 (2.4) [1.6-3.5]
Gastric	22 (2.0) [1.3-3.1]
Hepatobiliary	30 (2.8) [1.9-4.0]
Pancreatic	55 (5.1) [3.9-6.6]
Colorectal	64 (6.0) [4.6-7.5]
Other	23 (2.1) [1.4-3.2]
Lung	139 (12.9) [11.0-15.1]
Breast	118 (11.0) [9.2-13.0]
Genitourinary	89 (8.3) [6.7-10.1]
Gynecologic	80 (7.4) [5.9-9.2]
Hematologic-leukemia	72 (6.7) [5.3-8.4]
Lymphoma	72 (6.7) [5.3-8.4]
Prostate	57 (5.3) [4.0-6.8]
Hematologic-myeloma	47 (4.4) [3.2-5.8]
Head and neck	40 (3.7) [2.7-5.0]
Dermatologic	31 (2.9) [2.0-4.1]
CNS	29 (2.7) [1.8-3.8]
Sarcoma	25 (2.3) [1.5-3.4]
Endocrine	16 (1.5) [0.8-2.4]
Pulmonary-other	10 (0.9) [0.4-1.7]
Other	10 (0.9) [0.4-1.7]
Hematologic-other	9 (0.8) [0.4-1.6]
Cardiac	0 (0.0) [0.0-0.3]
Missing	11 (1.0) [0.5-1.8]
Presence of advanced cancer	674 (62.7) [59.7-65.6]
Cancer-related therapies within the previous 30 d	
Traditional chemotherapy	465 (43.3) [40.3-46.3]
Targeted drug therapy	193 (18.0) [15.7-20.4]
Systemic corticosteroids	162 (15.1) [13.0-17.4]
Radiotherapy	100 (9.3) [7.6-11.2]
Surgery for cancer	82 (7.6) [6.1-9.4]
None of the above	281 (26.1) [23.5-28.9]
Patient report living will or advanced directive	
None	492 (45.8) [42.8-48.8]
Yes	
Full code	193 (18.0) [15.7-20.4]
Do not resuscitate	161 (15.0) [12.9-17.2]
Do not intubate	6 (0.6) [0.2-1.2]
Comfort care only	28 (2.6) [1.7-3.7]
Other	114 (10.6) [8.8-12.6]
Unknown	81 (7.5) [6.0-9.3]
Patient report currently receiving palliative care	
Yes	86 (8.0) [6.4-9.8]
No	927 (86.2) [84.0-88.2]
Missing	62 (5.8) [4.4-7.3]
Patient report of currently receiving hospice care	
Yes	20 (1.9) [1.1-2.8]
No	1044 (97.1) [95.9-98.0]
Missing	11 (1.0) [0.5-1.8]

Abbreviation: CNS, central nervous system.

^a^ Cancer type, advanced cancer, and cancer-related therapy data are based on results of medical record review.

In the ED, pain was present in 668 patients (62.1%; 95% CI, 59.2%-65.0%), with shortness of breath (370 [34.4%; 95% CI, 31.6%-37.3%]), nausea (336 [31.3%; 95% CI, 28.5%-34.1%]), and abdominal pain (343 [31.9%; 95% CI, 29.1%-34.8%]) the next most common symptoms (Table 4); 249 patients (23.2%; 95% CI, 20.7%-25.8%) had no pain assessment documented in the ED medical record. Mean (SD) pain score among those with pain was 6.4 (2.6). More than one-third (386 [35.9%; 95% CI, 33.0%-38.8%]) reported moderate or severe pain in the ED. Opioids were administered to 381 participants (35.4%; 95% CI, 32.6%-38.4%), including 55 of 156 (35.2%; 95% CI, 27.8%-43.3%) with mild, 53 of 117 (45.3%; 95% CI, 36.1%-54.8%) with moderate, and 195 of 269 (72.5%; 95% CI, 66.7%-77.7%) with severe pain. Of the 386 participants with moderate or severe pain, 228 (59.1%; 95% CI, 18.8%-23.8%) received an opioid in the ED. Antiemetics were administered in 260 (24.2%; 95% CI, 21.3%-26.9%). Of those with nausea, 160 (47.6%; 95% CI, 12.8%-17.1%) received antiemetics in the ED.

**Table 4.** Symptoms and Symptom Treatment in 1075 Patients With Active Cancer Presenting to the ED

Symptoms and Symptom Treatment	Frequency, No. (%) [95% CI]
Patient reported symptoms on ED survey	
Any pain	668 (62.1) [59.2-65.0]
Shortness of breath	370 (34.4) [31.6-37.3]
Abdominal pain	343 (31.9) [29.1-34.8]
Nausea	336 (31.3) [28.5-34.1]
Chest pain	169 (15.7) [13.6-18.0]
Urinary symptoms	158 (14.7) [12.6-17.0]
Pain severity in the ED (score)^a^	
None	284 (26.4) [23.8-29.2]
Mild (1-4)	156 (14.5) [12.4-16.8]
Moderate (5-6)	117 (10.9) [9.1-12.9]
Severe (7-10)	269 (25.0) [22.4-27.7]
Not documented	249 (23.2) [20.7-25.8]
Administration of any ED pain medication	519 (48.3) [45.2-51.3]
Type of pain medications administered in the ED	
Nonsteroidal anti-inflammatory drug, all types	62 (5.8) [4.4-7.3]
Acetaminophen (alone or as part of a combination)	164 (15.3) [13.2-17.5]
Tramadol hydrochloride	13 (1.2) [0.6-2.0]
Short-acting opioid or narcotic	337 (31.3) [28.6-34.2]
Long-acting opioid	76 (7.1) [5.6-8.8]
Other or unknown	19 (1.8) [1.1-2.7]
Any opioid administered in the ED	381 (35.4) [32.6-38.4]
Pain medication administration in patients with moderate or severe pain in the ED (n = 386)	
Nonsteroidal anti-inflammatory drug, all types	33 (8.5) [2.1-4.3]
Acetaminophen (alone or as part of a combination product)	69 (17.9) [5.0-8.0]
Tramadol hydrochloride	10 (2.6) [0.4-1.7]
Short-acting opioid or narcotic	202 (52.3) [16.5-21.2]
Long-acting opioid	46 (11.9) [3.1-5.7]
Other or unknown	5 (1.3) [0.2-1.1]
Any opioid administered in the ED	228 (59.1) [18.8-23.8]
Nausea control documented in the ED medical record	
Any antiemetic administered in the ED	260 (24.2) [1.6-3.5]
Patients with nausea in the ED (n = 336)	160 (47.6) [12.8-17.1]

Abbreviation: ED, emergency department.

^a^ Initial mean (SD) pain score among the 542 patients with pain was 6.4 (2.6).

In the week before the ED visit, 302 participants (28.1%; 95% CI, 25.4%-30.9%) had no performance deficit based on Eastern Cooperative Oncology Group score (Table 1). For the Functional Assessment of Cancer Therapy–General reporting prior week symptoms, 776 participants (72.2%; 95% CI, 69.4%-74.8%) reported pain and 368 (47.4%; 95% CI, 31.4%-37.2%) of these had outpatient opioid prescriptions. This included 428 (39.8%; 95% CI, 36.9%-42.8%) participants with quite a bit or very much pain, 244 (57.0%; 95% CI, 52.2%-61.8%) of whom had outpatient opioid prescriptions. Half of all participants (541 [50.3%; 95% CI, 47.3%-53.4%]) reported nausea, with 359 of these having home nausea medications (66.4%; 95% CI, 62.2%-70.3%). Also, 214 participants (19.9%; 95% CI, 17.6%-22.4%) reported quite a bit or very much nausea, with 161 (75.2%; 95% CI, 68.9%-80.9%) of these having taken nausea medications at home. Similar rates of quite a bit or very much symptoms were due to lack of energy (584 [54.3%; 95% CI, 51.3%-57.3%]), worry that the condition will worsen (360 [33.5%; 95% CI, 30.1%-36.4%]), poor sleep (408 [38.0%; 95% CI, 35.0%-40.9%]), decreased enjoyment of life (469 [43.6%; 95% CI, 40.6%-46.6%]), and decreased quality of life (409 [38.0%; 95% CI, 35.1%-41.0%]).

Table 5 shows suspected infection. Fever immediately before presentation or in the ED was present in 155 (14.4%; 95% CI, 12.4%-16.7%) and neutropenia in 26 (2.4%). Antibiotics were administered to 285 participants (26.5%; 95% CI, 23.9%-29.2%). Blood and urine cultures were obtained in 255 patients (23.7%; 95% CI, 21.2%-26.4%), and blood cultures yielded positive findings in 27 (2.5%; 95% CI, 1.7%-3.6%) and urine cultures in 54 (5.0%; 95% CI, 3.8%-6.5%). Participants with suspected infection as identified by ED antibiotic administration were admitted at higher rates than those not receiving antibiotics (209 of 285 [73.3%; 95% CI, 17.1%-21.9%] vs 427 of 790 [54.1%; 95% CI, 50.5%-57.6%]), with 19.1% (40 of 209) having an LOS of no more than 2 days. Although some antibiotic recipients probably did not have acute bacterial infections, the paradigm of timely antibiotic administration in at-risk populations without a priori evidence of acute bacterial infection accounts for the antibiotic use observed in this population where 465 participants (43.3%; 95% CI, 40.3%-46.3%) recently received chemotherapy.

**Table 5.** Characteristics of 1075 Patients With Active Cancer and Suspected Infection Presenting to the ED

Characteristic	Frequency, No. (%) [95% CI]
Patient report of fever within 24 h before ED presentation^a^	120 (11.2) [9.3-13.2]
Temperature ≥38.0°C documented in the ED	88 (8.2) [6.6-10.0]
Fever reported at home or documented in the ED	155 (14.4) [12.4-16.7]
Neutropenia (neutrophil count <500/μL) present	26 (2.4) [1.6-3.5]
ED blood culture findings	
Positive	27 (2.5) [1.7-3.6]
Negative	228 (21.2) [18.8-23.8]
Not ordered or missing	820 (76.3) [73.6-78.8]
ED urine cultures	
Positive for >10 000 pathogenic organisms	54 (5.0) [3.8-6.5]
Contaminant growth reported by laboratory	65 (6.0) [4.7-7.6]
Negative	187 (17.4) [15.2-19.8]
Not ordered	772 (71.8) [69.0-74.5]
Patient taking antibiotics at home	147 (13.7) [11.7-15.9]
Antibiotics administered in the ED	285 (26.5) [23.9-29.2]
Admission among subjections receiving antibiotics (n = 285)	209 (73.3) [17.1-21.9]
Length of stay ≤2 d among those receiving antibiotics and admitted (n = 209)	40 (19.1) [2.7-5.0]

Abbreviation: ED, emergency department.

SI conversion factor: To convert neutrophil count to ×10^9^ per liter, multiply by 0.001.

^a^ Indicates temperature 38.0°C or higher.

Missing values are noted in Table 1 and Table 3. Interrater reliability was measured in 115 medical records (eTable 2 in the Supplement). Pain scores and worst vital signs had κ ≥ 0.80. Presence of advanced cancer, recent cancer therapy, and comorbidities had κ ≥ 0.60 (with most κ ≥ 0.80), except stroke (κ = 0.56) and recent corticosteroid administration (κ = 0.50).

## Discussion

The growing ED population of patients with active cancer^2,8^ present while receiving cancer therapy and with high acuity, high symptom burden, and frequent need for admission. This analysis provides the epidemiologic foundation to pursue care improvement opportunities, notably risk stratification, symptom management, and disposition in this population. These opportunities extend beyond the ED to include a need for timely specialty consultation, adequate outpatient follow-up to facilitate ED discharges, consistently applied processes targeting commonly encountered issues, and improved outpatient management to prevent ED visits.

We have identified a need for improved symptom control before and during the ED visit. Pain is a driver of ED presentation among patients with cancer,^4,12^ and appropriately managing pain is a significant contributor to improving quality of life in these patients.^25,26^ Pain in the ED was present in 62.1% of study participants, whereas 249 (23.2%; 95% CI, 20.7%-25.8%) had no pain assessment documented in the ED medical record. Nevertheless, a significant percentage of participants with moderate or severe pain did not receive opioids in the ED. This finding may reflect unexpected efficacy of nonnarcotic pain medications or an undesirable effect of current efforts to decrease overall ED opioid administration. Moreover, a significant portion of study patients were receiving outpatient opioid regimens, likely necessitating larger doses of opioids to effectively treat acute pain because opioid-tolerant ED patients with cancer frequently receive inadequate initial opioid doses.^27^ The pervasiveness of pain among patients presenting to the ED with active cancer underscores the need to recognize potential opioid tolerance and to explore less conventional ED pain control modalities (eg, subdissociative-dose ketamine and intravenous lidocaine).^28^

Poorly controlled pain was also frequently present in the week before the ED visit, with almost half of those reporting quite a bit or very much pain not receiving outpatient opioids. A significant opportunity therefore exists to improve pain control in the ED and outpatient settings, which could improve quality of life and avoid ED visits and hospital admissions. Improvements could include pragmatically designed clinical pathways that are primarily developed by emergency medicine physicians, in collaboration with oncology and palliative care colleagues, to improve symptom control and ensure adequate administration of analgesia. Given the low proportion of patients receiving palliative care services at time of ED visit (8.0%), the opportunity for additional intervention in these patients before prior to the ED visit is substantial.

Dyspnea and nausea were frequently present. Dyspnea is a particularly worrisome and challenging symptom because it may represent many causes of life-threatening pathologic conditions (eg, acute coronary syndrome, pulmonary embolism, pneumonia, cardiac tamponade, pleural effusion, pneumonitis) that, after disease progression, are leading causes of death in patients with cancer.^29^ The frequent presence of dyspnea as well as the broad differential diagnosis of life-threatening conditions likely contributes to high testing and admission rates. For nausea, ED and outpatient treatment appear to be suboptimal. This finding provides additional opportunities to improve quality of life and outpatient and ED management and develop care plan processes facilitating outpatient rather than inpatient care.

Suspicion of bacterial infection was also common. Although only 14.4% of participants had a recent or an ED-measured fever, blood and urine cultures were obtained in 255 (23.7%; 95% CI, 21.2%-26.4%), and antibiotics were administered in 26.5%. Participants receiving antibiotics were admitted at a higher rate than those not receiving antibiotics. Although some antibiotic recipients probably did not have acute bacterial infections, the paradigm of timely antibiotic administration in at-risk populations without a priori evidence of acute bacterial infection accounts for the antibiotic use observed in this population where 465 participants (43.3%; 95% CI, 40.3%-46.3%) recently received chemotherapy. Although only a small percentage (2.4%) of our cohort were found to have febrile neutropenia (a rate similar to that in prior ED studies),^12^ concern for this condition and the urgency to minimize time to antibiotics for these participants may have increased empirical antibiotic administration rates in febrile individuals who were ultimately found not to have neutropenia.

The large proportion of participants with suspected infection and subsequent admission identifies a population that could benefit from improved processes of care to ensure appropriate resource use and antibiotic administration in patients with and without neutropenia who have cancer and suspected infection. Risk stratification tools for infection focus on febrile neutropenia with conflicting results when applied in the ED setting.^30,31,32,33^ However, these tools are rarely used by oncologists or emergency physicians.^34,35^ Moreover, most of the participants with fever and/or suspected infection did not have febrile neutropenia. Little evidence regarding evaluation and risk stratification of these patients is available, and this area requires further investigation.

Use of palliative services is essential to outpatient care of patients with active cancer but was rare in our cohort, with only 8.0% receiving palliative care. Fewer than half reported having an advance directive. These low proportions, juxtaposed with the illness severity and substantive symptom burden of this ED population in the ED and in the week before the ED visit, justify exploring the ED’s potential for facilitating linkage to palliative care. Such linkage could, in turn, decrease subsequent ED use and improve quality of life.^36,37,38,39^ In addition, emergency physicians may communicate the need for advanced directives to patients with cancer and their outpatient physicians. Opportunities may exist for hospice referral, given the 5.8% 30-day mortality of the cohort, with only 1.9% receiving hospice care at the time of ED visit.^40^

Among our cohort, the admission rate was 57.2%, similar rates in prior research in patients with cancer,^2,4,5^ and much higher than the overall 30% rate in the study EDs and the national 9.0% rate.^1^ Given the risks of cancer-related complications along with the high symptom and comorbidity burdens of the ED population with active cancer, this observation is not surprising. Population-based research has identified disposition of patients with cancer who present to the ED to be associated with cancer type, comorbidities, age, race, and insurance type.^41^ Still, for those patients admitted purely for symptom control, for unavailability of specialty consultation, or for minimal interventions (eg, intravenous fluids or waiting for culture results only), admission may be overused and constitute a risk (hospital-acquired infection, deep venous thrombosis, fall) that outweighs the benefit of inpatient management. The potential to affect admission decisions may be greatest among the 19.1% of admitted patients with lengths of stay of no longer than 2 days.

The Centers for Medicare & Medicaid Services^42^ established a quality metric in 2016 that identifies diagnoses associated with potentially preventable ED use and hospital admission in patients with cancer, including pain, fever, dehydration, nausea, and emesis, among others. Such diagnoses may be present in half of patients with cancer who present to the ED.^12^ However, because patients with these symptoms may have serious life-threatening conditions, additional work will be needed to develop risk stratification tools and modifications to ED and outpatient oncology processes that could provide an opportunity to safely decrease ED presentation and admission rates for patients with cancer. An additional area requiring study is the use of ED observation units to safely and efficiently treat this population. In addition, cancer center models, including infusion centers and the Oncology Medical Home Model,^43^ may provide similar benefit.

These identified areas represent areas of opportunity to better support patients through their cancer treatment. Use of the ED appears to frequently be a marker for poorly controlled symptoms. Ultimately, identifying those patients at risk of ED use for potentially preventable conditions would allow intervention in the outpatient setting before symptoms become poorly controlled, improving overall care of the patient with cancer as well as achieving better ED resource use. Reducing ED use will also require novel approaches by oncologists to improve the support, access, and coordination of care for patients with cancer. These approaches could include development of systems to allow for more aggressive and preemptive management of expected complications. Such support will have to be easily accessible and available after hours and on weekends. The support should include scheduled follow-up checks as well as provisions for unscheduled in-person and remote care. Solving the acute access problem for those who need contact with the medical system but may not require ED care will be a key challenge for oncologists.

In addition, given the frequency and predictability of these symptoms, the opportunity is available to assist with and improve ED care, particularly when the primary oncology team is unavailable to treat the patient. Creating diagnostic, risk stratification, and treatment algorithms in partnership among emergency medicine, oncology, palliative care, and other stakeholders is necessary. A critical factor to improving such systems will be ensuring appropriate and rapid communication and follow-up for the patient, because lack of patient information and concern about the post-ED course is one of the primary drivers for ED admission decisions.^44^

### Limitations

Our study has several limitations. First, this convenience sample included patients from large, primarily urban, academic medical centers, many affiliated with comprehensive cancer centers. Community hospital ED cancer care may differ in patient characteristics, resource availability, and outcomes.^45,46^ As a result, our findings should be considered representative of large academic cancer centers. However, as we note below, in administrative national and statewide data sets, admission rates were similar between our study and others.^2,4^

We also may have underestimated severity within the participating academic institutions themselves. Although only 135 participants (12.6%; 95% CI, 10.6%-14.7%) arrived on a weekend compared with 27% to 28% in administrative data sets, we enrolled 94 patients (8.7%; 95% CI, 7.1%-10.6%) arriving at night (11 pm-7 am) compared with approximately 13% in administrative data sets.^2,4^ Also, 162 of 2337 approached patients (6.9%) were too ill or otherwise able to participate. One hundred forty non–English-speaking patients were assessed for eligibility (6.0%) and constituted 18.1% of the 773 approached who were deemed ineligible. Because patients with cancer and limited English proficiency report inferior treatment outcomes, their ineligibility may have affected results.^47^ Any potential underestimated illness severity allows for 2 possible effects. First, our identified symptom severity and frequency admission rate, hospital length of stay, and other variables related to severity potentially represent the lower bound of severity. Second, underestimation of severity could result in a ceiling effect because it is more challenging to improve outcomes in a healthier compared with less healthy populations. Also, ED revisit and hospital readmission rates may be underestimated because we could not identify use of health care services in other hospitals systems.

Despite these concerns, findings in administrative data sets suggest that our study population was reasonably representative. Rivera et al^2^ examined ED visits among adults with cancer using the Nationwide Emergency Department Sample, which includes academic and community EDs, and Mayer et al^4^ examined administrative data, including all ED visits in North Carolina. Both studies found similar admission rates (59.7% and 63%, respectively) as our study and a similar distribution of cancer types, which should diminish concerns regarding underestimation of disease severity.

## Conclusions

This study represents, to our knowledge, the first prospective, multicenter investigation describing ED use by patients with cancer. By prospectively enrolling patients, we were able to identify in much greater detail several patient factors than in previous work. Most of the patients presenting to the ED with active cancer were ill on presentation and had poorly controlled symptoms. Most were admitted or placed in observation, many for short hospital stays. Our data suggest opportunities to improve care for patients before, during, and after an ED stay. Success will require goal-oriented collaboration among oncology, palliative care, and emergency medicine but will allow for improved ED use, improved symptom control and risk stratification in the ED, and establishment of safe disposition decisions for patients with cancer.

## Notes

Supplement. **eFigure.** Study Enrollment Diagram **eTable 1.** Characteristics of Participating Emergency Departments **eTable 2.** Interrater Reliability of Medical Record Review in 1075 Emergency Department Patients With Cancer
