# Supplementary material for: Analysis of Diagnoses, Symptoms, Medications, and Admissions Among Patients With Cancer Presenting to Emergency Departments
Source: JAMA Netw Open. 2019 Mar 22;2(3):e190979. doi: 10.1001/jamanetworkopen.2019.0979 (PMC6583275; doi:10.1001/jamanetworkopen.2019.0979)
Supplement: Supplement. — eFigure. Study Enrollment Diagram eTable 1. Characteristics of Participating Emergency Departments eTable 2. Interrater Reliability of Medical Record Review in 1075 Emergency Department Patients With Cancer [file jamanetwopen-2-e190979-s001.pdf]

Supplementary Online Content

Caterino JM, Adler D, Durham DD, et al. Analysis of diagnoses, symptoms, medications, and admissions among patients with cancer presenting to emergency departments. *JAMA Netw Open*. 2019;2(3):e190979. doi:10.1001/jamanetworkopen.2019.0979

eFigure. Study Enrollment Diagram

eTable 1. Characteristics of Participating Emergency Departments

eTable 2. Interrater Reliability of Medical Record Review in 1075 Emergency Department Patients With Cancer

This supplementary material has been provided by the authors to give readers additional information about their work.

© 2019 Caterino JM et al. *JAMA Network Open.*

eFigure. Study Enrollment Diagram

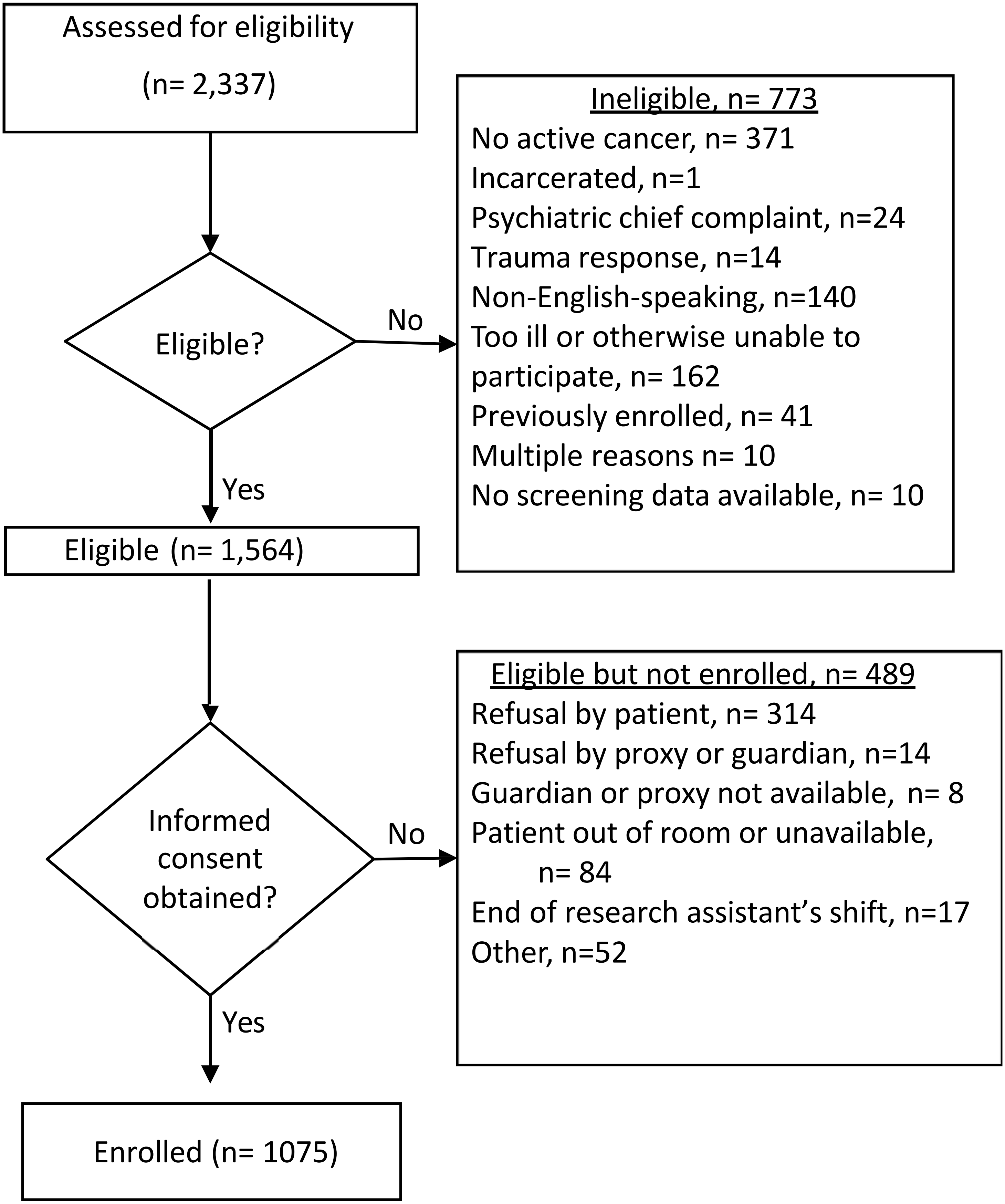

© 2019 Caterino JM et al. *JAMA Network Open.*

eTable 1. Characteristics of Participating Emergency Departments									
**Emergency Department**	**City, State**	**Type**	**Annual patient volume**	**ED ad-mission rate**	**ED active cancer patients***	**EM res-idency**	**Desig-nated cancer ED****	**Affiliated with NCI Comp-rehensive cancer center**	**Number subjects enrolled**
The Ohio State University Wexner Medical Center	Columbus, OH	Urban, Level 1 Trauma Center, Academic	72,000	30%	15%	Y	Y	Y	70
NYU Bellevue Hospital Center	New York, NY	Urban, Level 1 Trauma Center, Academic	125,000	20%	2%	Y	N	Y	18
NYU Langone Medical Center	New York, NY	Urban, Academic	65,000	30%	4%	Y	N	Y	69
UT MD Anderson Cancer Center	Houston, TX	Urban, Academic	20,000	40%	100%	Y	Y	Y	70
Tampa General Hospital	Tampa, FL	Urban, Level 1 Trauma Center, Academic	90,000	40%	6%	Y	N	Y	45
Beaumont Royal Oak	Royal Oak, MI	Suburban, Level 1 Trauma center, Community teaching	125,100	34%	5-10%	Y	N	N	70
Beaumont- Troy Hospital	Troy, MI	Suburban, Level II Trauma Center, Community teaching	85,000	34%	5-10%	Y	N	N	42
Brigham and Women's Hospital	Boston, MA	Urban, Level 1 Trauma Center, Academic	60,050	29%	10%	Y	N	Y	71
Yale University	New Haven, CT	Urban, Level 1 Trauma Center, Academic	90,000	30%	2%	Y	N	Y	72
Allegheny General Hospital	Pittsburgh, PA	Urban, Level 1 Trauma Center, Academic	55,000	24%	1.5%	Y	N	N	22
University of California-San Diego	San Diego, CA	Urban, Level 1 Trauma Center, Academic	80,000	20%	10%	Y	N	Y	48
Memorial Sloan Kettering Cancer Center	New York, NY	Urban, Academic	24,000	47%	95%	N	Y	Y	71
Beth Israel Deaconess Medical Center	Boston, MA	Urban, Level 1 Trauma Center, Academic	55,000	30%	15%	Y	N	Y	60
Saint Vincent Hospital	Worcester, MA	Urban, Level 2 Trauma Center, Community teaching	75,000	35%	15	Y	N	N	70
University of Washington	Seattle, WA	Urban, Level 1 Trauma Center, Academic	30,000	30%	10%	Y	N	Y	70
University of Rochester Medical Center	Rochester, NY	Urban, Level 1 Trauma Center, Academic	116,000	26%	4%	Y	N	N	71
University of Utah	Salt Lake City, UT	Urban, Level 1 Trauma Center, Academic	50,000	30%	5%	Y	N	Y	66
University of Cincinnati	Cincinnati, OH	Urban, Level 1 Trauma Center, Academic	76,800	20%	1.5%	Y	N	N	70
*Median proportion of cancer visits 8% (interquartile range 4-15%); mean proportion 17% (standard deviation 29%); **ED sees only patients with cancer (MSK/MD) or has a designated area specifically for cancer patients (OSU)									

© 2019 Caterino JM et al. *JAMA Network Open.*

eTable 2. Interrater Reliability of Medical Record Review in 1075 Emergency Department Patients With Cancer	
**Variable**	**Kappa**
Initial pain score	0.779
Worst temperature	0.7991
Worst systolic blood pressure	0.8479
Worst heart rate	0.9435
Worst respiratory rate	0.9395
Worst pain score	0.8481
Final pain score	0.7943
Myocardial infarction	1
Congestive heart failure	0.9119
Cerebrovascular accident	0.5554
Chronic obstructive pulmonary disease	0.6176
Diabetes	0.9263
Diabetes with end-organ damage	0.6624
Metastases	0.648
Mild liver disease	0.8289
Severe liver disease	0.9148
Chemotherapy in the past 30 days	0.7391
Targeted drug therapy in the past 30 days	0.6764
Systemic steroids in the past 30 days	0.4967
Radiation therapy in the past 30 days	0.6825
Surgery for cancer in the past 30 days	0.7313
Advanced cancer	0.6195
Note: Worst vital sign values were dichotomized as normal/abnormal as defined prior to abstraction	

© 2019 Caterino JM et al. *JAMA Network Open.*
